# The Protective Effect of Kaempferol Against Ischemia/Reperfusion Injury Through Activating SIRT3 to Inhibit Oxidative Stress

**DOI:** 10.21470/1678-9741-2020-0549

**Published:** 2022

**Authors:** Chuang Sun, Tingting Wang, Changying Wang, Zhenyin Zhu, Xiaoni Wang, Jia Xu, Huixian An

**Affiliations:** 1 Heart Hospital, Xi’an International Medical Center Hospital, Xi’an, People’s Republic of China.

**Keywords:** Kaempferols, Reperfusion Injury, Sirtuin-3, Oxidative Stress

## Abstract

**Introduction:**

The objective of this study is to investigate the protective effect of kaempferol against ischemia/reperfusion (IR) injury and the underlying molecular mechanisms.

**Methods:**

H9C2 cells were pretreated with kaempferol for 24 hours and further insulted with IR injury. Cell vitality, reactive oxygen species (ROS) level, glutathione (GSH) level, nicotinamide adenine dinucleotide phosphate (NADPH) oxidase activity, and sirtuin-3 (SIRT3), B-cell lymphoma 2 (Bcl2), and Bcl2-associated X protein (Bax) expressions were evaluated. Moreover, short interfering ribonucleic acid targeting SIRT3 was used to investigate the role of SIRT3 against IR mediated by kaempferol *in vitro*. IR mice models were also established to confirm the protective effects of kaempferol on IR *in vivo*.

**Results:**

After IR injury, H9C2 cells vitality was reduced, ROS levels, NADPH oxidase activity, and Bax expressions were increased, and GSH levels and Bcl2 expressions were decreased. After kaempferol pretreatment, the vitality of H9C2 cells was increased. The levels of ROS, NADPH oxidase activity, and Bax expression were decreased. In addition, levels of GSH and Bcl2 expression were enhanced. Furthermore, silencing SIRT3 attenuated the protective effect mediated by kaempferol, with increased ROS levels, NADPH oxidase activity, and Bax expression, along with reduced GSH level and Bcl2 expression. *In vivo* IR model showed that kaempferol could preserve IR-damaged cardiac function.

**Conclusion:**

Kaempferol has the capability of attenuating H9C2 cells IR injury through activating SIRT3 to inhibit oxidative stress.

**Table:** 

Abbreviations, acronyms & symbols				
Bax	= Bcl2-associated X protein		NADPH	= Nicotinamide adenine dinucleotide phosphate
Bcl2	= B-cell lymphoma 2			
CCK8	= Cell counting kit 8		ROS	= Reactive oxygen species
DMEM	= Dulbecco's Modified Eagle's Medium		SDS-PAGE	= Sodium dodecyl sulfate-polyacrylamide gel electrophoresis animation
GSH	= Glutathione		siRNA	= Short interfering ribonucleic acid
IR	= Ischemia/reperfusion		SIRT3	= Sirtuin-3
Kae	= Kaempferol		SOD2	= Superoxide dismutase
LVEF	= Left ventricular ejection fraction		SPSS	= Statistical Package for the Social Sciences
LVFS	= Left ventricular fractional shortening		USA	= United States of America

## INTRODUCTION

In recent decades, although many important progresses, including percutaneous coronary stent implantation and coronary thrombolysis, have been made in the treatment of myocardial ischemia, this disease is still a major life-threatening illness and brings a heavy economic burden worldwide^[1,2]^. The pathological mechanisms of the disease are associated with ischemia and hypoxia of local myocardial tissue, which can cause hypoxic injury of myocardial cells. In addition, the oxygen-free radicals in myocardial cells could explode after blood supply recovery and further aggravate the myocardial injury^[1]^. Therefore, it is very important to alleviate ischemia/reperfusion (IR) injury to better treat myocardial ischemia.

Kaempferol, also known as kaempferol-3, is a flavonoid compound that belongs to an active ingredient of traditional Chinese medicine. It has been reported that the compound has many physiological functions^[3-14]^, including anti-inflammatory^[3,4]^ and antitumor effects^[5-7]^ and protection against organ ischemic injury^[8-11]^. In addition, the protective effect of kaempferol in myocardial IR injury has also been confirmed^[9,11]^, but the associated molecular mechanisms are still unclear.

Sirtuin-3 (SIRT3) is a conserved nicotinamide adenine dinucleotide-dependent deacetylase that highly expresses in the myocardium. The reported studies show that SIRT3 has a significant antioxidative stress effect through deacetylating various mitochondrial-related proteins, such as superoxide dismutase 2 (SOD2)^[15,16]^. Marfe et al.^[17]^ found that, in the leukemia K562 cell line, kaempferol plays an antitumor role by regulating SIRT3. However, the protective effect of kaempferol and its regulatory effect on SIRT3 during myocardial IR remain unclear. In this study, the IR injury model was established, and the anti-IR injury effect of kaempferol and the molecular mechanisms associated with SIRT3 were explored.

## METHODS

### Cells and Reagents

Rat immortalized myocardial H9C2 cell line was purchased from the Shanghai cell bank of Chinese Academy of Sciences; kaempferol was from Sigma-Aldrich Company (United States of America [USA]); cell counting kit 8 (CCK8) was obtained from Qihai Biology Company (Shanghai, China); Dulbecco’s Modified Eagle’s Medium (DMEM), trypsin, penicillin, and streptomycin from Hyclone Company (USA); fetal bovine serum, protein quantitative kit, and Lipofectamin 3000 transfection reagent were collected from Thermo Fisher Scientific Inc. (USA); SIRT3 short interfering ribonucleic acid (siRNA) was designed and synthesized by Gemma company (Shanghai, China); cell culture bottles, Petri dishes, and centrifuge tubes were brought from Corning company (USA); SIRT3, SOD2, B-cell lymphoma 2 (Bcl2), BCL2-associated X protein (Bax), and β-actin antibodies were from Cell Signaling Technology (USA); goat anti-mouse and goat anti-rabbit second antibodies were obtained from Zhongshan Jinqiao Biological Company (Beijing, China); reactive oxygen species (ROS), glutathione (GSH), and nicotinamide adenine dinucleotide phosphate (NADPH) oxidase kit were purchased from Nanjing Jiancheng Institute of Bioengineering (Nanjing, China); and Western blot equipment, light-emitting photography system was purchased from Bio-Rad Company (USA).

### Cell Culture

The H9C2 cells were cultured in DMEM. When the cell density reaches about 80%, they are digested, centrifuged, and suspended, then inoculated into a 96-well plate or cell culture flask for further treatment. The concentration of kaempferol was 1 and 5 µg/mL, respectively.

### Establishment of the IR Model in H9C2 Cells

After the H9C2 cells were inoculated into a 96-well plate or cell culture flask for 12 hours, they were placed in a hypoxia-reoxygenation box (hypoxia condition: 95% N2, 5% CO_2_, four hours; reoxygenation condition: 95% air, 5% CO_2_, four hours) to establish IR cellular model.

### siRNA Plasmid Transfection

Cells were inoculated into a 6-well plate with a density of 1*10^5^ cells/well. After adherence, SIRT3 siRNA plasmid was transfected with Lipofectamine 3000, according to the instruction. After the transfection with siRNA plasmid for 24 hours, the levels of SIRT3 gene and protein were detected.

### CCK8 Assay

Cells were firstly inoculated into a 96-well plate with 1^X^10^4^/well and treated with IR after adherence. After treatment with kaempferol, the supernatant was discarded, and 100 ml DMEM with 10 ml CCK8 staining solution was added into each well. Then, the cells were incubated at 37 ºC for two hours without light. Spectra max multifunctional enzyme label was used to detect the absorbance at 450 nm wavelength, which represented the relative viability of the target cells.

### Western Blot

After digestion and centrifugation, the target cells were decomposed on ice for 20 minutes by adding lysate (RIPA, protease inhibitor: phosphatase inhibitor = 8:1:1). Then the supernatant was centrifuged at 4 ºC for 20 minutes and collected. A bicinchoninic acid kit was used to quantify the concentration of the target proteins. The supernatant was then mixed with the loading buffer and boiled for eight minutes. Sodium dodecyl sulfate-polyacrylamide gel electrophoresis animation (SDS-PAGE) was prepared according to the instructions, and the protein samples were added into the SDS-PAGE. After electrophoresis, the protein was transferred to polyvinylidene fluoride membrane, and the milk was used to block for two hours, and incubated overnight with the corresponding antibody at 4 ºC. Then the second antibody was incubated at room temperature for one hour. Finally, Bio-Rad chemiluminescence acquisition system was used to detect and analyze the protein expression.

### ROS Assay

The tests were carried out strictly according to the instructions. Briefly, the target cells were cultured with 10 µM dichloro-dihydro-fluorescein-diacetate for one hour at 37°C, and then the optical absorbance at the wavelength of 525 nm was measured, and ROS levels were calculated following the manual.

### GSH Detection

After digested, the target cells were treated with the GSH detecting system according to the instructions, the absorbance at 420 nm wavelength was detected, and the GSH content in cells was further calculated according to the formula in the instructions.

### Detection of NADPH Oxidase Activity

The cells were digested and then incubated with 250 µM/ml NADPH, according to the operation manual. The optical absorbance at the wavelength of 340 nm was detected, and NADPH oxidase activity was further calculated.

### Establishment and Treatment of Mice Models with IR

Animal experiments have been approved by the Ethics Committee of the Fourth Military Medical University. Thirty C57BL/6J mice were purchased from the Animal Center of Fourth Military Medical University. The animals were randomly divided into the control group, IR group, and IR + kaempferol (10 mg/kg) group. According to methods reported by previous research, the IR model of the myocardium in mice was established^[18]^. Ultrasound cardiac function was measured four hours after IR.

### Statistical Analysis

The Statistical Package for the Social Sciences (SPSS Inc. Released 2009, PASW Statistics for Windows, Version 18.0, Chicago: SPSS Inc.) software was used for statistical analysis. All data were expressed by mean with standard error of the mean. One-way analysis of variance was used for comparison between different groups, and *P*<0.05 was considered as statistically significant.

## RESULTS

### Effect of Kaempferol Pretreatment on the Viability of H9C2 Cells with IR

As shown in Table 1, the H9C2 cells were divided into the control group, IR group, IR + 1 µM kaempferol group, and IR + 5 µM kaempferol group. Cell viability was measured by the CCK8 method. The cell viability significantly decreased after IR treatment, compared to the control group (*P*<0.05). However, after pretreatment with 1 and 5 µM kaempferol, the cell viability increased in the IR group (*P*<0.05).

**Table 1:** Effects of kaempferol on H9C2 cell vitality with IR injury (%, x ± s).

Groups	Cell vitality
Control group	100±6.4
IR group	42.8±4.5^a^
IR + 1 µM kaempferol group	63.4±7.6^b^
IR + 5 µM kaempferol group	79.2±8.3^b^^c^

IR=ischemia/reperfusion Data were expressed as mean ± standard error of the mean (n=6).

a *P*<0.05 compared with the control group.

b *P*<0.05 compared with the IR group.

c *P*<0.05 compared with the IR + 5 µM kaempferol group.

### Effect of Kaempferol Pretreatment on Oxidative Stress Levels of H9C2 Cells with IR

**Fig. 1:**
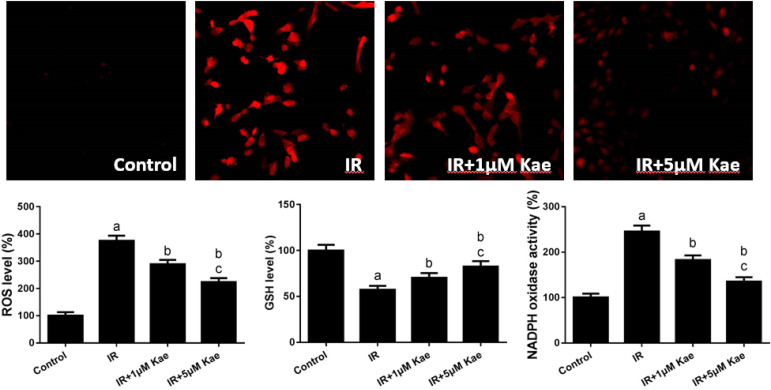
Effects of kaempferol (Kae) pretreatment on H9C2 cell oxidative stress after ischemia/reperfusion (IR) injury. Representative images of reactive oxygen species (ROS) staining were shown. Data were expressed as mean ± standard error of the mean (n=6). aP<0.05 compared with the control group; bP<0.05 compared with the IR group; cP<0.05 compared with the IR + 5 µM Kae group; GSH=glutathione; NADPH=nicotinamide adenine dinucleotide phosphate.

Current studies indicate that IR could enhance the oxidative stress levels in myocardial cells, and we were interested in exploring whether kaempferol treatment could inhibit the levels of oxidative stress in myocardial cells with IR. As shown in Figure 1, compared with the control group, IR treatment significantly activated the oxidative stress levels in H9C2 cells, which was characterized by an increase in ROS content, a decrease in GSH level, and an increase in NADPH oxidase activity. After pretreatment with 5 and 10 µM kaempferol, the level of oxidative stress decreased significantly, which was manifested by the decrease of ROS content, the raise of GSH level, and the decrease of NADPH oxidase activity in the IR group.

### Effect of Kaempferol Treatment on Expressions of SIRT3 and Apoptosis-Related Proteins of H9C2 Cells with IR

**Fig. 2:**
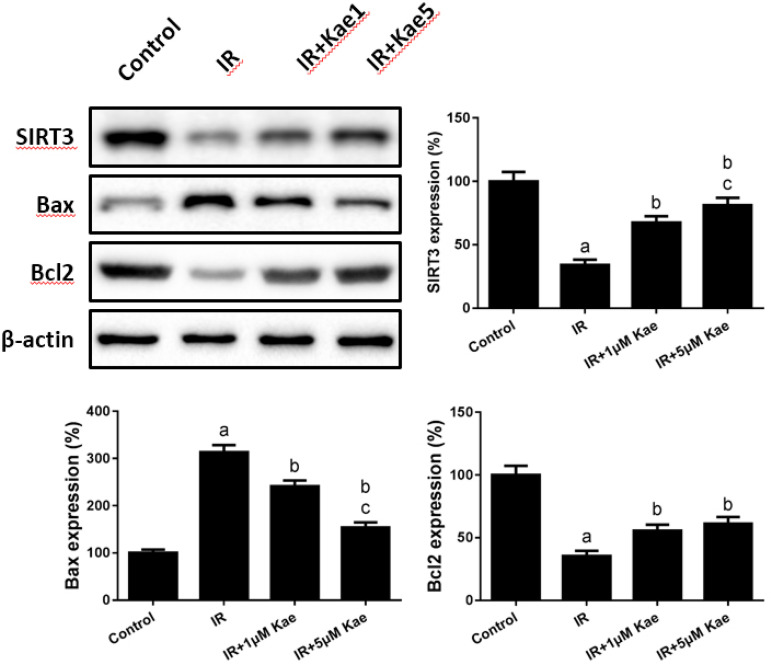
Roles of kaempferol (Kae) on H9C2 cell sirtuin-3 (SIRT3) expression and apoptosis level after ischemia/reperfusion (IR) injury. Data were expressed as mean ± standard error of the mean (n=6). aP<0.05 compared with the control group; bP<0.05 compared with the IR group; cP|<0.05 compared with the IR + 5 µM Kae group; Bax=Bcl2-associated X protein; Bcl2=B-cell lymphoma 2.

The current studies showed that SIRT3 has a significant anti-ROS effect, which is associated with the changes in apoptosis-related proteins^[19]^. In this study, we sought to investigate whether the anti-ROS effect mediated by kaempferol was associated with SIRT3 in myocardial cells. As shown in Figure 2, the expression of SIRT3 and antiapoptotic protein Bcl2 decreased significantly after IR treatment, while the expression of apoptotic protein Bax increased significantly (*P*<0.05). However, pretreatment with 1 and 5 µM kaempferol increased the expressions of SIRT3 and Bcl2 but inhibited the expression of Bax in H9C2 cells (*P*<0.05).

### Effects of SIRT3 on the Viability of H9C2 Cells with IR Mediated by Kaempferol

Next, we explored whether SIRT3 was involved in the increased viability of H9C2 cells with IR injury mediated by kaempferol. The cells were divided into the IR group, IR + SIRT3 siRNA group, IR + kaempferol group, and IR + SIRT3 siRNA + kaempferol group. As shown in Table 2, SIRT3 siRNA treatment had no significant effect on the viability of control cells (*P*>0.05). However, SIRT3 siRNA treatment significantly decreased cell viability (*P*<0.05) in the IR + kaempferol group, suggesting that the protective effect of kaempferol was dependent on SIRT3 in H9C2 cells.

**Table 2:** Co-treatment of SIRT3 siRNA and kaempferol on H9C2 cell vitality with IR injury (%).

Groups	Vitality
IR group	100±7.1
IR + siRNA group	95.6±7.5
IR + kaempferol group	187.2±11.3
IR + siRNA + kaempferol group	142.5±9.4^a^

IR=ischemia/reperfusion; siRNA=short interfering ribonucleic acid; SIRT3=sirtuin-3 Data were expressed as mean ± standard error of the mean (n=6).

a *P*<0.05 compared with IR + kaempferol group.

Effects of SIRT3 on Oxidative Stress Levels of H9C2 Cells with IR Mediated by Kaempferol

**Fig. 3:**
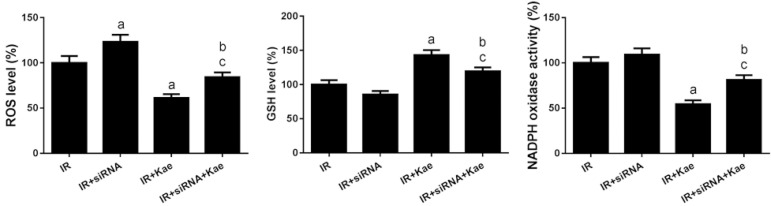
Co-treatment of sirtuin-3 short interfering ribonucleic acid (siRNA) and kaempferol (Kae) on H9C2 cell oxidative stress after ischemia/ reperfusion (IR). Data were expressed as mean ± standard error of the mean (n=6). aP<0.05 compared with the IR group; bP<0.05 compared with the IR + siRNA group; cP<0.05 compared with the IR + Kae group; GSH=glutathione; NADPH=nicotinamide adenine dinucleotide phosphate; ROS=reactive oxygen species.

As shown in Figure 3, SIRT3 siRNA treatment had no significant effect on oxidative stress levels of H9C2 cells with IR treatment. However, the oxidative stress levels increased significantly after the downregulation of SIRT3 expression in H9C2 cells in the IR + kaempferol group, with ROS content and NADPH oxidase activity increased, and GSH level decreased (*P*<0.05).

### Effects of SIRT3 on the Expression of Apoptotic-Related Proteins Mediated by Kaempferol in H9C2 Cells with IR

**Fig. 4:**
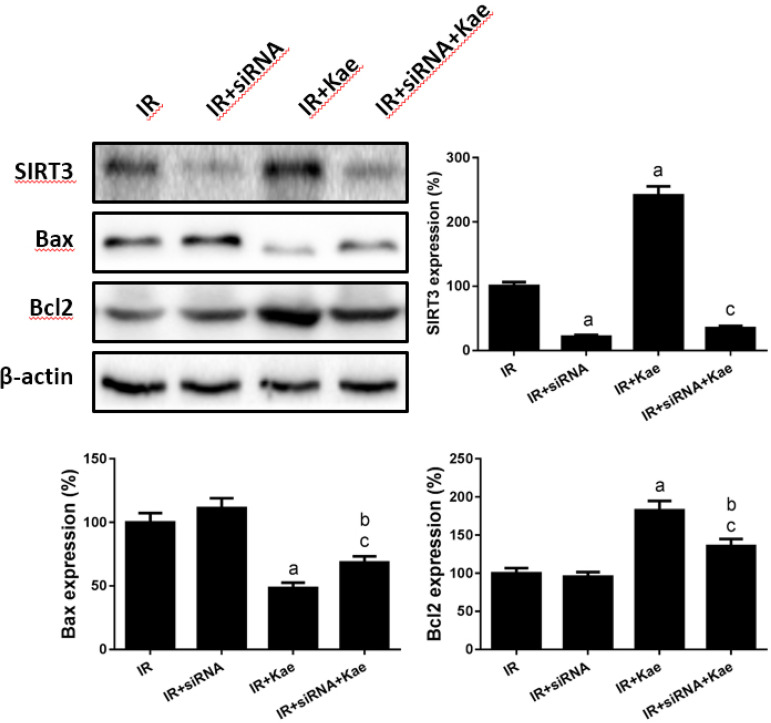
Co-treatment of sirtuin-3 (SIRT3) short interfering ribonucleic acid (siRNA) and kaempferol (Kae) on expression of SIRT3 and cell apoptosisrelated proteins. Data were expressed as mean ± standard error of the mean (n=6). aP<0.05 compared with the ischemia/reperfusion (IR) group; bP<0.05 compared with the IR + siRNA group; cP<0.05 compared with the IR + Kae group; Bax=Bcl2-associated X protein; Bcl2=B-cell lymphoma 2.

As shown in Figure 4, the expression of SIRT3 decreased significantly when mediated by SIRT3 siRNA treatment; the changes of Bcl2 and Bax expression were not obvious in H9C2 cells treated with IR. However, the expression of Bcl2 decreased but Bax increased after SIRT3 siRNA treatment in H9C2 cells in the IR + kaempferol group. These results suggested that SIRT3 played an important role in the expression of apoptotic-related proteins in H9C2 cells with IR (*P*<0.05).

Effect of Kaempferol Pretreatment on IR in Mice

**Fig. 5:**
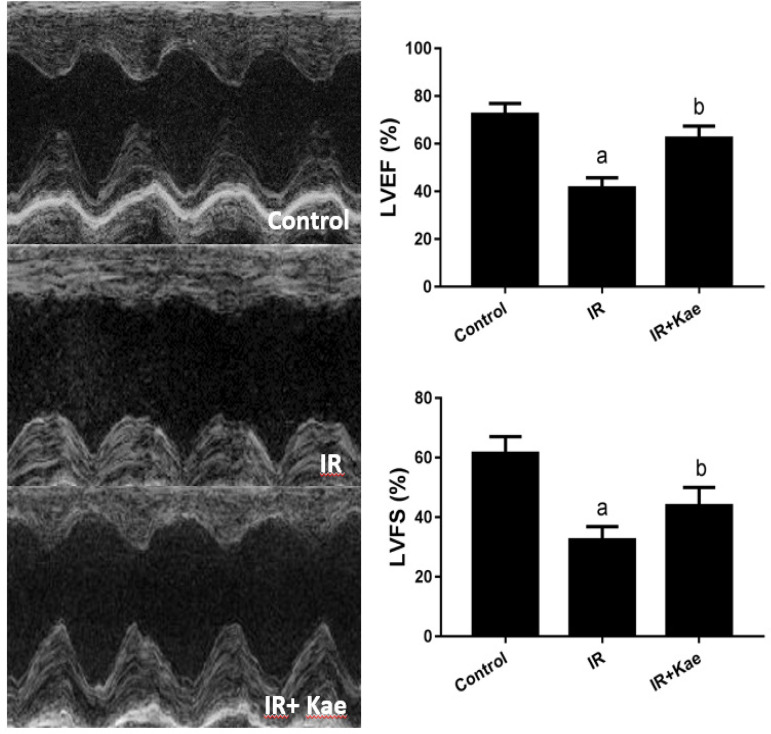
Effects of kaempferol (Kae) pretreatment on cardiac function with ischemia/reperfusion (IR) injury. Representative images of echocardiography were shown. Data were expressed as mean ± standard error of the mean (n=10). aP<0.05 compared with the control group; bP<0.05 compared with the IR group; LVEF=left ventricular ejection fraction; LVFS=left ventricular fractional shortening.

We also investigated the protective effect of kaempferol *in vivo* using the mice model, with detection of left ventricular ejection fraction (LVEF) and left ventricular fractional shortening (LVFS) levels by cardiac ultrasonography. The mice were divided into the control group, IR group, and IR + kaempferol (10 mg/kg) group. As shown in Figure 5, after IR treatment, the LVEF in mice was decreased significantly, compared to the control group (*P*<0.05). After pretreatment with 10 mg/kg kaempferol, the LVEF in mice with IR was increased (*P*<0.05). In addition, with the IR treatment, the LVFS value in mice decreased significantly. However, after pretreatment with 10 mg/kg kaempferol, the LVFS in mice with IR was increased (*P*<0.05). These results suggested that kaempferol had a protective effect on IR *in vivo*.

## DISCUSSION

The pathological mechanism of myocardial IR injury is related to ischemia and hypoxia of local myocardial tissue, which could cause hypoxic injury of myocardial cells. In addition, the oxygen-free radicals in myocardial cells explode after blood supply recovery, which further aggravates myocardial cell injury. Therefore, how to alleviate myocardial IR injury is a hot and difficult topic in the field of coronary heart disease clinical and experimental research. Most of the studies found that oxidative stress contributed to the development of IR^[20-22]^. Oxidative stress could cause mitochondrial dysfunction, increase ROS synthesis, decrease GSH level, increase NADPH reductase activity, and activate downstream apoptotic pathway^[23-25]^. Kaempferol is a flavonoid derivative with multiple biological effects. In recent years, it has been found that kaempferol mediates the protective effects of IR injury in the heart, brain, kidney, and other organs^[15,26,27]^. In this study, we explore the effect of kaempferol on IR injury, and the results confirmed that kaempferol could effectively alleviate the damage of H9C2 cells induced by IR. Chen et al.^[28]^ have found that kaempferol can resist to lipopolysaccharide-induced myocardial injury *in vitro* and *in vivo*, and its molecular mechanism is related to the inhibition of oxidative stress. In our study, we observed that the oxidative stress levels in H9C2 cells increased after IR injury. After kaempferol treatment, oxidative stress indicators, ROS, and NADPH oxidase activity decreased significantly, and GSH increased significantly in *in vitro* models. These results suggested that kaempferol may play a role in myocardial protection by inhibiting oxidative stress stimulation.

SIRT3 is a major mitochondrial deacetylase, which plays an important role in maintaining mitochondrial function and regulating the level of oxidative stress in cells^[29]^. Current reports have confirmed that activating SIRT3-related signaling pathways can significantly increase IR injury in the heart and brain^[20,30]^. However, whether SIRT3 was involved in the cardioprotective effect mediated by kaempferol was unknown. In the study, we detected the expression of SIRT3 in H9C2 cells after IR treatment and found that the expression of SIRT3 was significantly decreased. However, SIRT3 expression increased significantly after kaempferol treatment, accompanied by oxidative stress and the decline of apoptotic levels. These results implied that SIRT3 may be essential for the protective role mediated by kaempferol in IR. To further clarify the key role of SIRT3 in kaempferol's anti-IR injury, we used siRNA to downregulate the expression of SIRT3 and then applied IR treatment. The results showed that the protective effect of kaempferol was weakened, and the levels of oxidative stress and apoptosis were also increased after the expression of SIRT3 was knocked down. Together, these results suggest that SIRT3, as an important regulator of oxidative stress, is involved in the cardioprotective effect of kaempferol.

## CONCLUSION

In summary, this study clarified the protective effect of kaempferol on IR injury of H9C2 cells and mice *in vivo*, and the SIRT3/oxidative stress pathway was involved in this process. Our findings could provide a theoretical reference for the development of cardioprotective drugs based on kaempferol.

**Table:** 

Authors' roles & responsibilities	
CS	Substantial contributions to the conception or design of the work; or the acquisition, or analysis of data for the work; drafting the work or revising it critically for important intellectual content; agreement to be accountable for all aspects of the work in ensuring that questions related to the accuracy or integrity of any part of the work are appropriately investigated and resolved; final approval of the version to be published
TW	Substantial contributions to the conception of the work; drafting the work; final approval of the version to be published
CW	Substantial contributions to the acquisition of data for the work; final approval of the version to be published
ZZ	Substantial contributions to the acquisition of data for the work; final approval of the version to be published
XW	Substantial contributions to the acquisition of data for the work; final approval of the version to be published
JX	Substantial contributions to the acquisition or analysis of data for the work; final approval of the version to be published
HA	Substantial contributions to the conception of the work; or the acquisition of data for the work; drafting the work or revising it critically for important intellectual content; agreement to be accountable for all aspects of the work in ensuring that questions related to the accuracy or integrity of any part of the work are appropriately investigated and resolved; final approval of the version to be published
